# Association of Preoperative Renin-Angiotensin System Inhibitors With Prevention of Postoperative Atrial Fibrillation and Adverse Events: A Systematic Review and Meta-analysis

**DOI:** 10.1001/jamanetworkopen.2019.4934

**Published:** 2019-05-31

**Authors:** Shaojie Chen, Willem-Jan Acou, Marcio G. Kiuchi, Christian Meyer, Philipp Sommer, Martin Martinek, Alexandra Schratter, Bruno R. Andrea, Zhiyu Ling, Shaowen Liu, Yuehui Yin, Gerhard Hindricks, Helmut Pürerfellner, Mitchell W. Krucoff, Boris Schmidt, K. R. Julian Chun

**Affiliations:** 1 Cardioangiologisches Centrum Bethanien, Frankfurt Academy for Arrhythmias, Medizinische Klinik III, Agaplesion Markus Krankenhaus, Frankfurt am Main, Germany; 2 Department of Cardiology, AZ Delta, Roeselare, Belgium; 3 School of Medicine, Royal Perth Hospital Unit, University of Western Australia, Perth; 4 Klinik für Kardiologie mit Schwerpunkt Elektrophysiologie, Universitäres Herzzentrum Hamburg, Universitätsklinikum Hamburg-Eppendorf, Hamburg; 5 German Centre for Cardiovascular Research, Partner Site Hamburg/Kiel/Lübeck, Germany; 6 Klinik für Elektrophysiologie/Rhythmologie, Herz- und Diabeteszentrum Nordrhein-Westfalen, Universitätsklinik der Ruhr-Universität Bochum, Bad Oeynhausen, Germany; 7 Abteilung der Elektrophysiologie, Herzzentrum Universität Leipzig, Leipzig, Germany; 8 Abteilung der Kardiologie, Akademisches Lehrkrankenhaus der Elisabethinen, Ordensklinikum Linz Elisabethinen, Linz, Austria; 9 Medizinische Abteilung mit Kardiologie, Krankenhaus Hietzing Wien, Vienna, Austria; 10 Department of Cardiology, Sul Fluminense University Hospital, Vassouras, Brazil; 11 Department of Cardiology, The Second Affiliated Hospital of Chongqing Medical University, Chongqing Cardiac Arrhythmia Therapeutic Service Center, Chongqing, China; 12 Department of Cardiology, Shanghai General Hospital, Shanghai Jiao Tong University School of Medicine, Shanghai, China; 13 Department of Medicine and Cardiology, Duke University Medical Center and Duke Clinical Research Institute, Durham, North Carolina

## Abstract

**Importance:**

Postoperative atrial fibrillation (POAF) is a well-known complication after cardiac surgery. Renin-angiotensin system inhibitors (RASIs) have been suggested as an upstream therapy for selected patients with AF; however, evidence in the surgical setting is limited.

**Objective:**

To evaluate the role of preoperative RASIs in prevention of POAF and adverse events for patients undergoing cardiac surgery.

**Data Sources:**

The PubMed database and the Cochrane Library from inception until December 31, 2018, were searched by using the keywords *renin-angiotensin system inhibitors* OR *angiotensin-converting enzyme inhibitors* OR *angiotensin receptor blocker* OR *aldosterone antagonist* AND *cardiac surgery*. ClinicalTrials.gov was searched from inception until December 31, 2018, by using the keywords *postoperative atrial fibrillation*.

**Study Selection:**

Randomized clinical trials (RCTs) and observational studies comparing the association between preoperative RASI treatment vs no preoperative RASI treatment (control group) and the incidence of POAF were identified. Eleven unique studies met the selection criteria.

**Data Extraction and Synthesis:**

Pooled analysis was performed using a random-effects model. Sensitivity and subgroup analyses of RCTs were performed to test the stability of the overall effect. Metaregression was conducted to explore potential risk of bias.

**Main Outcomes and Measures:**

The primary outcome was POAF, and the secondary outcomes included rates of stroke and mortality and duration of hospitalization.

**Results:**

Eleven unique studies involving 27 885 unique patients (74.4% male; median age, 65 years [range, 58.5-74.5 years]) were included. Compared with the control group, the RASI group did not have a significantly reduced risk of POAF (odds ratio [OR], 1.04; 95% CI, 0.91-1.19; *P* = .55; *z* = 0.60), stroke (OR, 0.86; 95% CI, 0.62-1.19; *P* = .37; *z* = 0.90; without significant heterogeneity, *P* = .11), death (OR, 1.07; 95% CI, 0.85-1.35; *P* = .56; *z* = 0.59; without significant heterogeneity, *P* = .12), composite adverse cardiac events (OR, 1.04; 95% CI, 0.91-1.18; *P* = .58; *z* = 0.56), or a reduced hospital stay (weighted mean difference, −0.04; 95% CI, −1.05 to 0.98; *P* = .94; *z* = 0.07) using a random-effects model. Pooled analysis focusing on RCTs showed consistent results. The primary overall effect was maintained in sensitivity and subgroup analyses. Metaregression showed that male sex was significantly associated with POAF (τ^2^ = 0.0065; *z* = 3.47; *Q* = 12.047; *P* < .001) and that use of β-blockers was associated with a significantly reduced risk in developing POAF (τ^2^ = 0.018; *z* = −2.24; *Q* = 5.0091; *P* = .03).

**Conclusions and Relevance:**

The findings from this study suggest that preoperative RASI treatment does not offer additional benefit in reducing the risk of POAF, stroke, death, and hospitalization in the setting of cardiac surgery. The results provide no support for conventional use of RASIs for the possible prevention of POAF and adverse events in patients undergoing cardiac surgery; further randomized data, particularly among those patients with heart failure, are needed.

## Key Points

**Question:**

Does the use of renin-angiotensin system inhibitors have any association with reduction in atrial fibrillation and adverse events for patients undergoing cardiac surgery?

**Findings:**

This systematic review and meta-analysis involving 11 unique studies with 27 885 patients undergoing cardiac surgery found no additional association between preoperative renin-angiotensin system inhibitor therapy and a reduced risk of postoperative atrial fibrillation, stroke, death, or hospitalization.

**Meaning:**

The results provide no support for the routine use of renin-angiotensin system inhibitors for the possible prevention of postoperative atrial fibrillation and adverse events in patients undergoing cardiac surgery.

## Teaser

This systematic review and meta-analysis evaluates the association of preoperative renin-angiotensin system inhibitors with prevention of postoperative atrial fibrillation and adverse events among patients undergoing cardiac surgery.

## Introduction

Postoperative atrial fibrillation (POAF) is a well-known complication of cardiac surgery, putting patients at an increased risk of morbidity and mortality. Epidemiological studies^1,2,3,4,5^ showed that approximately one-third of patients develop atrial fibrillation (AF) after coronary artery bypass graft surgery (CABG), meaning that of the approximately 800 000 people who undergo CABG worldwide, more than 264 000 will develop post-CABG AF. Large-sample data analysis also showed that men had a significantly higher risk of POAF.^1,2,3,4,5^

POAF is also costly in terms of medical resources owing to the prolonged duration of hospitalization.^6,7^ Results from a 2014 large-sample study^8^ showed that POAF is strongly associated with long-term development of AF after cardiac surgery.

Advanced age, history of AF, metabolic syndrome, and cardiac and renal dysfunction have been found to be potential risk factors for POAF, but the underlying mechanisms of POAF remain elusive.^9^ Research^9^ has suggested that inflammation, dysregulation of the autonomic nervous system, and overactivation of the renin-angiotensin system (RAS) system are involved in the pathogenesis of POAF.

Previous data^10,11^ have shown that use of RAS inhibitors (RASIs), that is, upstream therapy, was associated with reduced risk of AF in selected populations because of their organ protection, remodeling prevention, and anti-inflammation effects. However, the existing consensus is mainly derived from nonsurgical populations, and the role of preoperative RASI therapy among patients in the surgical setting remains unclear.^12^ In this study, we aim to evaluate the role of preoperative use of RASIs for the prevention of POAF in patients undergoing cardiac surgery by conducting a collaborative systematic review and meta-analysis of the most updated clinical studies.

## Methods

This study was performed in accordance with the Preferred Reporting Items for Systematic Reviews and Meta-analyses (PRISMA) reporting guideline for conducting a systematic review and meta-analysis.^13^ Each individual study included in the systematic review was approved by their own institutional ethics committee, and the informed consent was obtained by each individual study.

### Search Strategies

We identified clinical trials that compared the preoperative use of RASIs (treatment group) with no RASIs (control group) in patients undergoing cardiac surgery. An online search of the published literature was performed using the PubMed database and the Cochrane Library from inception until December 31, 2018. The search was developed by using keywords *renin-angiotensin system inhibitors* OR *angiotensin-converting enzyme inhibitors* OR *angiotensin receptor blocker* OR *aldosterone antagonist* AND *cardiac surgery*. An additional search was performed in the ClinicalTrials.gov website from inception until December 31, 2018, using keywords *postoperative atrial fibrillation*.

### Selection Criteria and Study Outcomes

Randomized and observational studies were included in the initial search. The treatment group comprised patients treated with RASIs, including angiotensin-converting enzyme inhibitors, angiotensin receptor blockers, or aldosterone antagonists, whereas the control group comprised patients who did not receive any RASIs. The study population consisted of patients undergoing CABG or cardiac valvular surgery. The primary outcome was rate of POAF, the identification of which was based on 12-lead electrocardiographic monitoring during the study period. The rates of stroke and mortality and duration of hospitalization were analyzed as secondary outcomes. The selection criteria were predefined, and the search process was conducted by 2 investigators (S.C. and M.G.K.). Discrepancies were resolved by discussions.

### Data Extraction and Quality Assessment

Data extraction and presentation followed the recommendations of the PRISMA statement.^13^ The data extracted included study characteristics, patient characteristics, treatment, and outcome data. We assessed the methodological quality of the included studies based on the recommendation of the PRISMA statement^13^ and the *Cochrane Handbook for Systematic Reviews of Interventions*.^14^ The key items were summarized by using the following queries: (1) randomized study? (2) double-blinded? (3) clear definition of study population? (4) clear definition of study comparison? (5) clear definition of outcome assessment? (6) appropriate statistical method used? (7) no selective loss of data analysis? and (8) important confounders identified? Each yes response scored 1 point, and a study assessed with 6 points or more was considered high quality. Discrepancy regarding study quality was resolved by consensus.

### Statistical Analysis

The statistical analyses were performed following the recommendations of the *Cochrane Handbook for Systematic Reviews of Interventions.*^14^ The categorical variables were reported as percentages and estimated using a Mantel-Haenszel odds ratio (OR) with a 2-tailed 95% CI, whereas continuous variables were analyzed by weighted mean difference. The *I*^2^ statistic assessed by *Q* test was used to quantify the degree of between-study heterogeneity. Given the intrinsic variations in study design, we calculated the OR or the weighted mean difference estimates using random-effects models for all comparisons. Overall effect of primary outcome was tested by sensitivity analysis.

Publication bias was assessed using the Begg adjusted rank correlation test and the Egger regression asymmetry test.^14^ To explore the effect of covariates on the overall treatment effect, we performed a random-effects meta-regression analysis, wherein the logarithm of the OR for primary outcome was regressed against the baseline characteristics of the included studies. All *P* values were 2 tailed, and the statistical significance was set at *P* < .05. Statistical analyses were performed using the Revman software, version 5.3 (The Cochrane Collaboration); Stata software, version 12.0 (StataCorp); and Comprehensive Meta-Analysis Software, version 2.2 (Biostat, Inc).

## Results

### Baseline Characteristics

As shown in eFigure 1 in the Supplement, the search strategy retrieved a total of 2045 citations. Of these, 1758 reports were excluded based on titles, abstracts, and article types. The remaining 287 full-text articles were screened further for eligibility, and 276 were excluded through predefined inclusion criteria. Consequently, 11 unique studies characterizing a total of 27 885 unique patients (median age, 65 years [range, 58.5-74.5 years]; 74.4% male and 25.6% female) were selected into our meta-analysis.^15,16,17,18,19,20,21,22,23,24,25^ The baseline characteristics of included studies are summarized in Table 1, and quality assessment for included trials is presented in eTable 1 in the Supplement. Outcome data of included studies were extracted and are detailed in eTable 2 in the Supplement. eTable 3 in the Supplement summarizes the use of RASIs in individual studies. eTable 4 in the Supplement shows the perioperative use of antiarrhythmic drugs.

**Table 1.** Baseline Characteristics of Individual Studies

Source	Study Design	Study Setting	Intervention of Interest	Sample Size, RASI/Control Groups, No. of Participants	Mean Age, RASI/Control Groups, y	Male sex, RASI/Control Groups, %	Prior AF, RASI/Control Groups, %	EH, RASI/Control Groups, %	Diabetes, RASI/Control Groups, %	Prior MI, RASI/Control Groups, %	Use of β-Blockers, RASI/Control Groups, %	Use of Statins, RASI/Control Groups, %	CHF, RASI/Control Groups, %	Length of Follow-up
White et al,^15^ 2007	PC	CABG/VS	Preoperative RASIs	175/163	65.4/65.9	76.6/79.1	4.0/3.1	89.7/65.6	45.7/22.7	42.9/31.9	82.3/85.9	76.0/60.1	12.6/7.4	30 d
Ozaydin et al,^16^ 2008	RCT	CABG/VS	Preoperative RASIs	98/30	58.5/60.0	55.1/70.0	Excluded	61.2/40.0	39.8/36.7	NA	88.8/96.7	NA	9.2/6.7	In hospital
Miceli et al,^17^ 2009	RC	CABG	Preoperative ACEI	3052/3052	64.9/64.8	80.5/80.7	3.0/3.3	67.8/68.7	14.4/14.1	46.4/46.0	NA	NA	NA	30 d
Rader et al,^18^ 2010	RC	CABG/VS	Preoperative RASIs	3437/3437	66/66	72.4/74.8	Excluded	86/70	45/30	NA	68/66	NA	39/18	In hospital
Yoo et al,^19^ 2010	RC	CABG	Preoperative RASIs	296/176	65/65	63.9/56.8	NA	68.6/61.4	43.6/31.8	NA	69.3/64.8	51/46	NA	30 d
El-Haddad et al,^20^ 2011	RCT	CABG	Preoperative ARB	50/50	58.7/60.0	58/52	Excluded	80/88	58/52	NA	82/82	60/62	NA	In hospital
Radaelli et al,^21^ 2011	RC	CABG	Preoperative RASIs	1635/1504	61.2/61.6	64.2/68.9	4.1/2.8	83.2/60.9	34.3/24.5	46.9/36.2	NA	NA	46.9/36.2	In hospital
Barodka et al,^22^ 2011	RC	CABG/VS	Preoperative RASIs	122/224	73.8/74.5	71/62	NA	90.2/75.9	32.8/27.2	NA	81.2/74.6	73.3/70.2	14.8/8.0	30 d
Bandeali et al,^23^ 2012	RC	CABG	Preoperative ACEI	3983/4906	64.2/63.6	74.2/76.4	6.7/7.1	93.7/76.5	49.3/33.1	42.2/39.7	59.0/50.8	71.9/57.9	NA	In hospital
Chin et al,^24^ 2012	RC	CABG	Preoperative RASIs	407/643	64/65	74.7/75.3	Excluded	74.2/56.1	53.8/41.7	20.9/15.9	63.6/59.6	65.8/58.3	2.9/1.6	In hospital
Pretorius et al,^25^ 2012	RCT	CABG/VS	Preoperative RASIs	298/147	58.9/60.0	67.7/64.0	Excluded	61.1/66.0	22.1/20.4	NA	50.3/49.0	53.0/57.1	NA	In hospital

Abbreviations: ACEI, angiotensin-converting enzyme inhibitor; AF, atrial fibrillation; ARB, angiotensin receptor blocker; CABG, coronary artery bypass graft; CHF, congestive heart failure; EH, essential hypertension; MI, myocardial infarction; NA, not available; PC, prospective controlled; RASI, renin-angiotensin system inhibitor; RC, retrospective controlled; RCT, randomized clinical trial; VS, valvular surgery.

### Primary Outcome for RASIs and POAF

**Figure 1.**
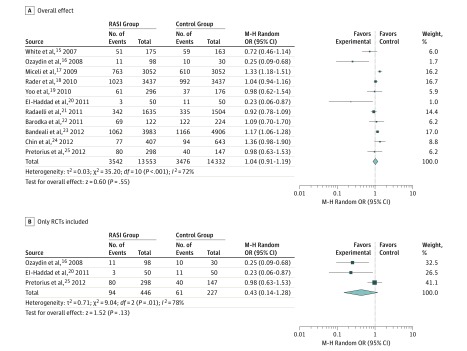
Pooled Analysis for the Comparison of the Risk for Postoperative Atrial Fibrillation (POAF) M-H indicates Mantel-Haenszel; OR, odds ratio; and RASI, renin-angiotensin system inhibitor.

All included studies reported POAF events. A total of 13 553 participants were allocated to the RASI group and 14 332 to the control group. As demonstrated in Figure 1A, the incidence of POAF was 3542 (26.1%) in the RASI group and 3476 (24.3%) in the control group. Use of RASIs was not associated with reduced risk of POAF (OR, 1.04; 95% CI, 0.91-1.19; *P* = .55; *z* = 0.60, random-effects model). A notable between-study heterogeneity was observed in this comparison (*I*^2^ = 72%).

### Meta-Analysis Focusing on Randomized Trials

Three randomized clinical trials (RCTs) were included in the systematic review.^16,20,25^ Pooled analysis of these RCTs did not show a significant association of RASI use over the control group with POAF (OR, 0.43; 95% CI, 0.14-1.28; *P* = .13; *z* = 1.52, with random-effects model) (Figure 1B).

### Sensitivity Analysis and Subgroup Analyses

Results of the Begg (τ = −0.47; *z* = 2.02; *P* = .04) and Egger (intercept, −1.66 [SE, 0.79]; *t* = 2.08; *P* = .07) tests showed a moderate risk of publication bias. Sensitivity analysis further demonstrated a good stability of the overall effect (eFigure 2 in the Supplement). As shown in Table 2, subgroup analyses showed consistent results based on study types (OR for RCTs only, 0.43; 95% CI, 0.14-1.28; *P* = .13), study settings (OR for CABG setting only, 1.12 [95% CI, 0.95-1.33; *P* = .18]; OR for CABG and valvular surgery setting, 0.88 [95% CI, 0.67-1.17; *P* = .39]), sample size (OR for studies with >100 patients each group, 1.10; 95% CI, 0.98-1.22; *P* = .11), new-onset AF (OR, 0.87; 95% CI, 0.59-1.26; *P* = .44), and follow-up period (OR for in-hospital POAF, 1.02 [95% CI, 0.86-1.20; *P* = .84]; OR for 30-day POAF, 1.06 [95% CI, 0.80-1.41; *P* = .69]).

**Table 2.** Subgroup Analyses for Effect of RASIs on POAF (RASI vs Control Groups)

Subgroups Based on Study Characteristics	No. of Studies Included	Incidence of POAF, RASI/Control Groups, %	OR (95% CI)	*z* Effect	*P* Value
Only RCTs included	3	21.1/26.8	0.43 (0.14-1.28)	1.52	.13
Only CABG setting	6	24.5/21.8	1.12 (0.95-1.33)	1.34	.18
CABG and VS setting	5	29.9/30.1	0.88 (0.67-1.17)	0.87	.39
>100 Patients each group	9	26.3/24.2	1.10 (0.98-1.22)	1.62	.11
New-onset AF	5	27.8/26.6	0.87 (0.59-1.26)	0.47	.44
In-hospital POAF	7	26.2/24.7	1.02 (0.86-1.20)	0.20	.84
30-d POAF	4	25.9/22.9	1.06 (0.80-1.41)	0.41	.69
Use of β-blockers >80%	4	20.1/32.9	0.40 (0.17-0.94)	−2.11	.04
Participants >70% male	6	26.9/23.9	1.15 (1.01-1.32)	2.13	.03

Abbreviations: AF, atrial fibrillation; CABG, coronary artery bypass graft; OR, odds ratio; POAF, postoperative atrial fibrillation; RASI, renin-angiotensin system inhibitor; RCT, randomized clinical trial; VS, valvular surgery.

### Metaregression Analysis

**Figure 2.**
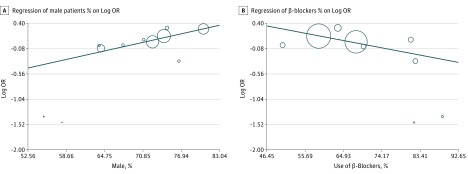
Regression Analyses of Male Sex and Use of β-Blockers Against Logarithm Odds Ratio (OR) Circles indicate the individual study included in the regression analysis; different sizes indicate the sample size of the individual study.

The results of regression analysis are demonstrated in Table 3. Known baseline variables for regression analysis included sample size, age, sex, history of AF, hypertension, diabetes, previous myocardial infarction, heart failure, use of a β-blocker, and use of statins. Male sex was significantly associated with an increased risk of POAF (τ^2^ = 0.0065; *z* = 3.47; *Q* = 12.047; *P* < .001), and use of β-blockers was significantly associated with a reduced risk of POAF (τ^2^ = 0.018; *z* = −2.24; *Q* = 5.0091; *P* = .03) (Figure 2).

**Table 3.** Metaregression Analyses

Covariates	No. of Observations	τ^2^ Value	*z* Value for Slope	*Q* Value	*P* Value	Significance With the Logarithm OR
Sample size	11	0.026	1.66	2.75	.10	No
Age	11	0.027	1.40	1.97	.16	No
Male sex	11	0.0065	3.47	12.047	<.001	Significant
Prior AF	4	0.072	0.064	0.0041	.95	No
EH	11	0.035	0.068	0.0047	.95	No
Diabetes	11	0.032	−0.82	0.67	.41	No
Prior MI	5	0.028	−0.84	0.71	.40	No
CHF	6	0.039	−0.059	0.0035	.95	No
β-Blockers	9	0.018	−2.24	5.0091	.03	Significant
Statins	7	0.042	0.20	0.041	.84	No

Abbreviations: AF, atrial fibrillation; CHF, congestive heart failure; EH, essential hypertension; MI, myocardial infarction; OR, odds ratio.

### Secondary Outcomes of Stroke and Mortality, and Composite Outcome of Adverse Cardiac Events

Five trials with 22 658 patients reported postoperative stroke events.^17,18,22,23,25^ Meta-analysis showed a similar incidence of stroke in the RASI and control groups (1.8% vs 2.0%; OR, 0.86; 95% CI, 0.62-1.19; *P* = .37; *z* = 0.90; without significant heterogeneity, *P* = .11) (eFigure 3 in the Supplement). Seven studies with a total of 26 269 patients reported data for all-cause mortality.^17,18,19,21,22,23,25^ The pooled result showed no significant difference regarding the mortality rate between the RASI and control groups (3.2% vs 3.2%; OR, 1.07; 95% CI, 0.85-1.35; *P* = .56; *z* = 0.59; without significant heterogeneity, *P* = .12) (eFigure 3 in the Supplement). Further meta-analysis for a composite outcome of adverse cardiac events showed similar rates of events between the RASI and control groups (30.6% vs 28.9%; OR, 1.04; 95% CI, 0.91-1.18; *P* = .58; *z* = 0.56) (eFigure 3 in the Supplement).

### Hospitalization

Four studies reported the outcome data of hospitalization.^19,20,22,25^ Pooled analysis of this outcome showed a similar duration of hospital stay between the RASI and control groups (weighted mean difference, −0.04; 95% CI, −1.05 to 0.98; *P* = .94; *z* = 0.07) (eFigure 4 in the Supplement).

## Discussion

### Main Findings

In this study, we compared the rate of POAF and adverse events in patients undergoing cardiac surgery who received preoperative RASI treatment vs a control group of patients undergoing cardiac surgery who did not receive RASI treatment. The results demonstrated a nonsignificant association of preoperative RASIs in reducing the risk of POAF; moreover, preoperative RASI treatment was not associated with a reduced rate of postoperative stroke, shortened hospitalization, or decreased perioperative mortality.

### Mechanism of Postoperative AF and Potential Role of RASIs in AF

POAF is a well-known clinical complication, and it occurs in approximately one-third of patients undergoing cardiac surgery.^26^ Despite remarkable development in medical care and technology during the last few decades, the incidence of POAF remains unsatisfactorily high, leading to significantly increased morbidity, health care costs, and even mortality.^1,2,3,4,5,6,7,8^ In our pooled data including 27 885 patients who underwent cardiac surgery, 7018 (25.2%) developed POAF, suggesting an urgent need for a therapeutic approach to treat this postoperative complication.

The specific mechanism underlying POAF remains to be fully elucidated. Structural and electrical remodeling has been proposed to be associated with the pathogenesis of POAF.^26^ Perioperative cardiac injuries and structural cardiac diseases (ie, atrial dilation and atrial fibrosis) are important elements of cardiac structural remodeling that can result in diverse electrical conduction properties and therefore configure new reentry foci. Pathophysiologically, ischemic myocardial damage, inflammatory response, and disturbance of autonomic tone contribute to cardiac electrical remodeling, and all these factors play a pivotal role in the initiation and maintenance of POAF.^26^ The development of POAF seems multifactorial. Clinical factors such as advanced age, male sex, history of AF, hypertension, diabetes, and decreased left ventricular function have all been identified as risk factors for POAF.^6,9,27^ Cumulative data from clinical studies suggests that RASIs play a protective role to prevent AF in selected patients owing to their upstream regulation and antiremodeling effect.^28,29^ However, these studies mainly focused on patients in the nonsurgical setting.

### Interpretation of Previous Trials and Results

Among the included studies, only 2^16,20^ showed favorable outcomes in patients undergoing preoperative RASI therapy. Although the randomized method was applied, the statistical power of these 2 studies was largely restricted by the small sample size. At the same time, 2 large-scale observational studies^17,23^ demonstrated a significantly higher risk of major cardiac adverse events, including POAF, among patients who received preoperative RASI treatment.

The study by Pretorius and colleagues^25^ is, to date and to our knowledge, the largest RCT on this topic. Four hundred forty-five patients scheduled for cardiac surgery were randomized into treatment with angiotensin-converting enzyme inhibitors, angiotensin receptor blockers, or placebo (control group). All patients were treated with conventional medical care guided by institutional protocol.^25^ Blood tests for angiotensin-converting enzyme activity and aldosterone concentrations were performed after randomization. POAF was confirmed by continuous electrocardiographic monitoring throughout the postoperative period until discharge. During in-hospital follow-up, no statistical difference was found among the 3 groups regarding the incidence of POAF.^25^

### Conventional Management for POAF

At present, pharmacotherapy is the primary preventive method to reduce the incidence of POAF. The most widely used medication is β-blockers. A recent meta-analysis of 2556 patients undergoing cardiac surgery^30^ demonstrated a significantly reduced risk of POAF associated with β-blocker therapy. This finding seems to be in line with our regression analysis results, which suggest that β-blockers play a protective role in reducing POAF. Current international guidelines recommend that in the absence of contraindications, β-blockers should be given to patients scheduled to undergo cardiac surgery (class I, level A).^31,32^ Regarding class III antiarrhythmic drugs, amiodarone hydrochloride and sotalol hydrochloride were shown to significantly decrease the incidence of POAF; however, owing to their potential adverse effects such as bradycardia, hypotension, and ventricular arrhythmias, particularly in patients with electrolyte disturbance, such drugs should be used with caution.^31,33^ In addition, nonantiarrhythmic drugs targeting the atrial substrate and inflammation process are of interest. Statins are well known to lower lipid levels, and they have also been shown to exert a variety of protective effects such as anti-inflammation, antioxidation, and regulation of endothelial function. Previous large-sample meta-analyses found a substantially reduced risk of POAF, stroke, and mortality associated with statin therapy in patients undergoing cardiac surgery.^34,35^

Polyunsaturated ω-3 fatty acids participate in the stabilizing of biological membrane and are known to mitigate inflammation and oxidative stress and to potentially prevent remodeling. However, a major RCT^36^ and a meta-analysis^37^ failed to exhibit additional benefit of polyunsaturated ω-3 fatty acids in reducing the risk of POAF.

Corticosteroids exert potent anti-inflammation effects. A previous meta-analysis^38^ showed that corticosteroid treatment was associated with a significant reduction in POAF. However, given the potential adverse effects on glucose metabolism, wound healing, and infection, conventional use of corticosteroids for POAF prevention remains controversial.

Colchicine has also been shown to suppress chemotactic factor, attenuate neutrophil activation, and modulate endothelial cell adhesion and migration to injured tissues.^39^ In 2014, the Colchicine for Prevention of the Postpericardiotomy Syndrome and Postoperative Atrial Fibrillation (COPPS-2) trial^40^ showed that preoperative colchicine treatment was associated with decreased risk of postpericardiotomy syndrome, but no association was found with incidence of POAF.

### Significance of the Present Study

Pooled analyses using data from relevant clinical studies are increasingly used in evidence-based medicine, and clinical evidence of high quality can be provided if potential bias is properly controlled for. In the present study, we conducted a predefined literature search, including 27 885 patients from 11 published studies, most of which were of medium to high methodological quality. We predefined the primary outcome as the rate of POAF, and our results showed that preoperative RASI therapy was not associated with additional clinical benefit in the setting of cardiac surgery. Despite notable heterogeneity among the included studies, further evaluation by sensitivity analysis and comprehensive subgroup and meta-regression analyses did not show fundamental shifting of the overall effect, indicating a good stability of the primary result.

Consistent with the primary outcome, the analysis of secondary end points, including the risk of postoperative stroke, death, composite cardiac adverse events, and hospitalization, also revealed neutral results. These findings suggest that preoperative RASI therapy may not confer an additional protective effect for POAF prevention. Moreover, the metaregression analysis showed that male sex is a significant risk factor for POAF, and the use of β-blockers was associated with a significantly reduced risk in developing POAF.

### Limitations

Although methodological efforts have been made to control for potential bias, limitations of the present study should be noted. This study was a meta-analysis of aggregate data rather than an individual patient-level study. One major limitation of the present study is a lack of RCTs. Most of the included trials were retrospective in nature; despite comprehensive subgroup analyses and regression analyses, the influence of unreported confounding factors cannot be fully ruled out. Limited data were available regarding the specific types and doses of RASIs prescribed, making it very difficult, if not impossible, to distinguish the drug-class effect and the dose effect in our analysis.

## Conclusions

This large-sample pooled analysis involving existing evidence suggests that preoperative treatment using RASIs does not appear to offer additional benefit in reducing the acute risk of POAF, stroke, mortality, and hospitalization in the setting of cardiac surgery. Male sex was found to be associated with an increased risk of developing POAF, and the use of β-blockers was associated with a significantly reduced risk of developing POAF.

## Notes

Supplement. **eFigure 1.** PRISMA Flow Diagram for Study Selection **eFigure 2.** Sensitivity Analysis **eFigure 3.** Comparison for Risk of Stroke, Mortality, and Composite Events **eFigure 4.** Comparison for Hospitalization **eTable 1.** Quality Assessment **eTable 2.** Summary of Outcome Data Reported in Included Studies **eTable 3.** Use of RASIs in Individual Studies **eTable 4.** Concomitant AADs Use **eReferences**

## References

[1] Filardo G, Damiano RJ Jr, Ailawadi G, Epidemiology of new-onset atrial fibrillation following coronary artery bypass graft surgery. Heart. 2018;104(12):-. doi:10.1136/heartjnl-2017-312150 29326112

[2] Filardo G, Ailawadi G, Pollock BD, Sex differences in the epidemiology of new-onset in-hospital post-coronary artery bypass graft surgery atrial fibrillation: a large multicenter study. Circ Cardiovasc Qual Outcomes. 2016;9(6):723-730. doi:10.1161/CIRCOUTCOMES.116.003023 27756797

[3] Lahtinen J, Biancari F, Salmela E, Postoperative atrial fibrillation is a major cause of stroke after on-pump coronary artery bypass surgery. Ann Thorac Surg. 2004;77(4):1241-1244. doi:10.1016/j.athoracsur.2003.09.077 15063244

[4] Villareal RP, Hariharan R, Liu BC, Postoperative atrial fibrillation and mortality after coronary artery bypass surgery. J Am Coll Cardiol. 2004;43(5):742-748. doi:10.1016/j.jacc.2003.11.023 14998610

[5] Mathew JP, Parks R, Savino JS, ; MultiCenter Study of Perioperative Ischemia Research Group Atrial fibrillation following coronary artery bypass graft surgery: predictors, outcomes, and resource utilization. JAMA. 1996;276(4):300-306. doi:10.1001/jama.1996.03540040044031 8656542

[6] Zimmer J, Pezzullo J, Choucair W, Meta-analysis of antiarrhythmic therapy in the prevention of postoperative atrial fibrillation and the effect on hospital length of stay, costs, cerebrovascular accidents, and mortality in patients undergoing cardiac surgery. Am J Cardiol. 2003;91(9):1137-1140. doi:10.1016/S0002-9149(03)00168-1 12714166

[7] Auer J, Weber T, Berent R, Ng CK, Lamm G, Eber B Postoperative atrial fibrillation independently predicts prolongation of hospital stay after cardiac surgery. J Cardiovasc Surg (Torino). 2005;46(6):583-588. 16424847

[8] Lee SH, Kang DR, Uhm JS, New-onset atrial fibrillation predicts long-term newly developed atrial fibrillation after coronary artery bypass graft. Am Heart J. 2014;167(4):593-600.e1. doi:10.1016/j.ahj.2013.12.010 24655710

[9] Echahidi N, Pibarot P, O’Hara G, Mathieu P Mechanisms, prevention, and treatment of atrial fibrillation after cardiac surgery. J Am Coll Cardiol. 2008;51(8):793-801. doi:10.1016/j.jacc.2007.10.043 18294562

[10] Belluzzi F, Sernesi L, Preti P, Salinaro F, Fonte ML, Perlini S Prevention of recurrent lone atrial fibrillation by the angiotensin-II converting enzyme inhibitor ramipril in normotensive patients. J Am Coll Cardiol. 2009;53(1):24-29. doi:10.1016/j.jacc.2008.08.071 19118720

[11] Yin Y, Dalal D, Liu Z, Prospective randomized study comparing amiodarone vs amiodarone plus losartan vs amiodarone plus perindopril for the prevention of atrial fibrillation recurrence in patients with lone paroxysmal atrial fibrillation. Eur Heart J. 2006;27(15):1841-1846. doi:10.1093/eurheartj/ehl135 16825288

[12] Kirchhof P, Benussi S, Kotecha D, 2016 ESC Guidelines for the management of atrial fibrillation developed in collaboration with EACTS. Europace. 2016;18(11):1609-1678. doi:10.1093/europace/euw295 27567465

[13] Liberati A, Altman DG, Tetzlaff J, The PRISMA statement for reporting systematic reviews and meta-analyses of studies that evaluate healthcare interventions: explanation and elaboration. BMJ. 2009;339:b2700. doi:10.1136/bmj.b2700 19622552 PMC2714672

[14] Higgins JPT, Green S, eds. *Cochrane Handbook for Systematic Reviews of Interventions* Version 5.1.0. The Cochrane Collaboration. https://handbook-5-1.cochrane.org/. Updated March 2011. Accessed December 14, 2018.

[15] White CM, Kluger J, Lertsburapa K, Faheem O, Coleman CI Effect of preoperative angiotensin converting enzyme inhibitor or angiotensin receptor blocker use on the frequency of atrial fibrillation after cardiac surgery: a cohort study from the atrial fibrillation suppression trials II and III. Eur J Cardiothorac Surg. 2007;31(5):817-820. doi:10.1016/j.ejcts.2007.02.010 17350856

[16] Ozaydin M, Dede O, Varol E, Effect of renin-angiotensin aldosteron system blockers on postoperative atrial fibrillation. Int J Cardiol. 2008;127(3):362-367. doi:10.1016/j.ijcard.2007.05.012 17692951

[17] Miceli A, Capoun R, Fino C, Effects of angiotensin-converting enzyme inhibitor therapy on clinical outcome in patients undergoing coronary artery bypass grafting. J Am Coll Cardiol. 2009;54(19):1778-1784. doi:10.1016/j.jacc.2009.07.008 19682819

[18] Rader F, Van Wagoner DR, Gillinov AM, Blackstone EH Preoperative angiotensin-blocking drug therapy is not associated with atrial fibrillation after cardiac surgery. Am Heart J. 2010;160(2):329-336.e1. doi:10.1016/j.ahj.2010.05.033 20691840 PMC2919305

[19] Yoo YC, Youn YN, Shim JK, Kim JC, Kim NY, Kwak YL Effects of renin-angiotensin system inhibitors on the occurrence of acute kidney injury following off-pump coronary artery bypass grafting. Circ J. 2010;74(9):1852-1858. doi:10.1253/circj.CJ-10-0261 20631451

[20] El-Haddad MA, Zalawadiya SK, Awdallah H, Role of irbesartan in prevention of post-coronary artery bypass graft atrial fibrillation. Am J Cardiovasc Drugs. 2011;11(4):277-284. doi:10.2165/11587160-000000000-00000 21623642

[21] Radaelli G, Bodanese LC, Guaragna JC, The use of inhibitors of angiotensin-converting enzyme and its relation to events in the postoperative period of CABG. Rev Bras Cir Cardiovasc. 2011;26(3):373-379. doi:10.5935/1678-9741.20110011 22086573

[22] Barodka V, Silvestry S, Zhao N, Preoperative renin-angiotensin system inhibitors protect renal function in aging patients undergoing cardiac surgery. J Surg Res. 2011;167(2):e63-e69. doi:10.1016/j.jss.2009.11.702 20189597

[23] Bandeali SJ, Kayani WT, Lee VV, Outcomes of preoperative angiotensin-converting enzyme inhibitor therapy in patients undergoing isolated coronary artery bypass grafting. Am J Cardiol. 2012;110(7):919-923. doi:10.1016/j.amjcard.2012.05.021 22727178

[24] Chin JH, Lee EH, Son HJ, Preoperative treatment with an angiotensin-converting enzyme inhibitor or an angiotensin receptor blocker has no beneficial effect on the development of new-onset atrial fibrillation after off-pump coronary artery bypass graft surgery. Clin Cardiol. 2012;35(1):37-42. doi:10.1002/clc.20991 22020954 PMC6652464

[25] Pretorius M, Murray KT, Yu C, Angiotensin-converting enzyme inhibition or mineralocorticoid receptor blockade do not affect prevalence of atrial fibrillation in patients undergoing cardiac surgery. Crit Care Med. 2012;40(10):2805-2812. doi:10.1097/CCM.0b013e31825b8be2 22824930 PMC3588582

[26] Shingu Y, Kubota S, Wakasa S, Ooka T, Tachibana T, Matsui Y Postoperative atrial fibrillation: mechanism, prevention, and future perspective. Surg Today. 2012;42(9):819-824. doi:10.1007/s00595-012-0199-4 22619000

[27] Banach M, Rysz J, Drozdz JA, Risk factors of atrial fibrillation following coronary artery bypass grafting: a preliminary report. Circ J. 2006;70(4):438-441. doi:10.1253/circj.70.438 16565561

[28] Savelieva I, Kakouros N, Kourliouros A, Camm AJ Upstream therapies for management of atrial fibrillation: review of clinical evidence and implications for European Society of Cardiology guidelines, part I: primary prevention. Europace. 2011;13(3):308-328. doi:10.1093/europace/eur002 21345926

[29] Savelieva I, Kakouros N, Kourliouros A, Camm AJ Upstream therapies for management of atrial fibrillation: review of clinical evidence and implications for European Society of Cardiology guidelines, part II: secondary prevention. Europace. 2011;13(5):610-625. doi:10.1093/europace/eur023 21515595

[30] Arsenault KA, Yusuf AM, Crystal E, Interventions for preventing post-operative atrial fibrillation in patients undergoing heart surgery. Cochrane Database Syst Rev. 2013;1(1):CD003611. 23440790 10.1002/14651858.CD003611.pub3 PMC7387225

[31] Camm AJ, Kirchhof P, Lip GY, ; ESC Committee for Practice Guidelines Guidelines for the management of atrial fibrillation: the Task Force for the Management of Atrial Fibrillation of the European Society of Cardiology (ESC) [published correction appears in *Europace*. 2011;13(7):1058]. Europace. 2010;12(10):1360-1420. doi:10.1093/europace/euq350 20876603

[32] Fuster V, Rydén LE, Cannom DS, 2011 ACCF/AHA/HRS focused updates incorporated into the ACC/AHA/ESC 2006 guidelines for the management of patients with atrial fibrillation: a report of the American College of Cardiology Foundation/American Heart Association Task Force on Practice Guidelines developed in partnership with the European Society of Cardiology and in collaboration with the European Heart Rhythm Association and the Heart Rhythm Society. J Am Coll Cardiol. 2011;57(11):e101-e198. doi:10.1016/j.jacc.2010.09.013 21392637

[33] Crystal E, Garfinkle MS, Connolly SS, Ginger TT, Sleik K, Yusuf SS Interventions for preventing post-operative atrial fibrillation in patients undergoing heart surgery. Cochrane Database Syst Rev. 2004;4(4):CD003611. 15495059 10.1002/14651858.CD003611.pub2

[34] Liakopoulos OJ, Choi YH, Haldenwang PL, Impact of preoperative statin therapy on adverse postoperative outcomes in patients undergoing cardiac surgery: a meta-analysis of over 30,000 patients. Eur Heart J. 2008;29(12):1548-1559. doi:10.1093/eurheartj/ehn198 18506053

[35] Liakopoulos OJ, Kuhn EW, Slottosch I, Wassmer G, Wahlers T Preoperative statin therapy for patients undergoing cardiac surgery. Cochrane Database Syst Rev. 2012;4(4):CD008493. doi:10.1002/14651858.CD008493.pub2 22513959

[36] Mozaffarian D, Marchioli R, Macchia A, ; OPERA Investigators Fish oil and postoperative atrial fibrillation: the Omega-3 Fatty Acids for Prevention of Post-operative Atrial Fibrillation (OPERA) randomized trial. JAMA. 2012;308(19):2001-2011. doi:10.1001/jama.2012.28733 23128104 PMC3694745

[37] Mozaffarian D, Wu JH, de Oliveira Otto MC, Fish oil and post-operative atrial fibrillation: a meta-analysis of randomized controlled trials. J Am Coll Cardiol. 2013;61(21):2194-2196. doi:10.1016/j.jacc.2013.02.045 23541970 PMC3697850

[38] Ho KM, Tan JA Benefits and risks of corticosteroid prophylaxis in adult cardiac surgery: a dose-response meta-analysis. Circulation. 2009;119(14):1853-1866. doi:10.1161/CIRCULATIONAHA.108.848218 19332460

[39] Van Wagoner DR Colchicine for the prevention of postoperative atrial fibrillation: a new indication for a very old drug? Circulation. 2011;124(21):2281-2282. doi:10.1161/CIRCULATIONAHA.111.057075 22105193 PMC3256985

[40] Imazio M, Brucato A, Ferrazzi P, ; COPPS-2 Investigators Colchicine for prevention of postpericardiotomy syndrome and postoperative atrial fibrillation: the COPPS-2 randomized clinical trial. JAMA. 2014;312(10):1016-1023. doi:10.1001/jama.2014.11026 25172965

