# Supplementary material for: Association of Preoperative Renin-Angiotensin System Inhibitors With Prevention of Postoperative Atrial Fibrillation and Adverse Events: A Systematic Review and Meta-analysis
Source: JAMA Netw Open. 2019 May 31;2(5):e194934. doi: 10.1001/jamanetworkopen.2019.4934 (PMC6547087; doi:10.1001/jamanetworkopen.2019.4934)
Supplement: Supplement. — eFigure 1. PRISMA Flow Diagram for Study Selection eFigure 2. Sensitivity Analysis eFigure 3. Comparison for Risk of Stroke, Mortality, and Composite Events eFigure 4. Comparison for Hospitalization eTable 1. Quality Assessment eTable 2. Summary of Outcome Data Reported in Included Studies eTable 3. Use of RASIs in Individual Studies eTable 4. Concomitant AADs Use eReferences [file jamanetwopen-2-e194934-s001.pdf]

Supplementary Online Content

Chen S, Acou W-J, Kiuchi MG, et al. Association of preoperative renin-angiotensin system inhibitors with prevention of postoperative atrial fibrillation and adverse events: a systematic review and meta-analysis. *JAMA Netw Open.* 2019;2(5):e194934. doi:10.1001/jamanetworkopen.2019.4934

eFigure 1. PRISMA Flow Diagram for Study Selection
eFigure 2. Sensitivity Analysis
eFigure 3. Comparison for Risk of Stroke, Mortality, and Composite Events
eFigure 4. Comparison for Hospitalization
eTable 1. Quality Assessment
eTable 2. Summary of Outcome Data Reported in Included Studies
eTable 3. Use of RASIs in Individual Studies
eTable 4. Concomitant AADs Use
eReferences

This supplementary material has been provided by the authors to give readers additional information about their work.

©2019 Chen S et al. *JAMA Network Open*

eFigure 1. PRISMA Flow Diagram for Study Selection

Supplemental online Figure 1
PRISMA Flow Diagram for study selection

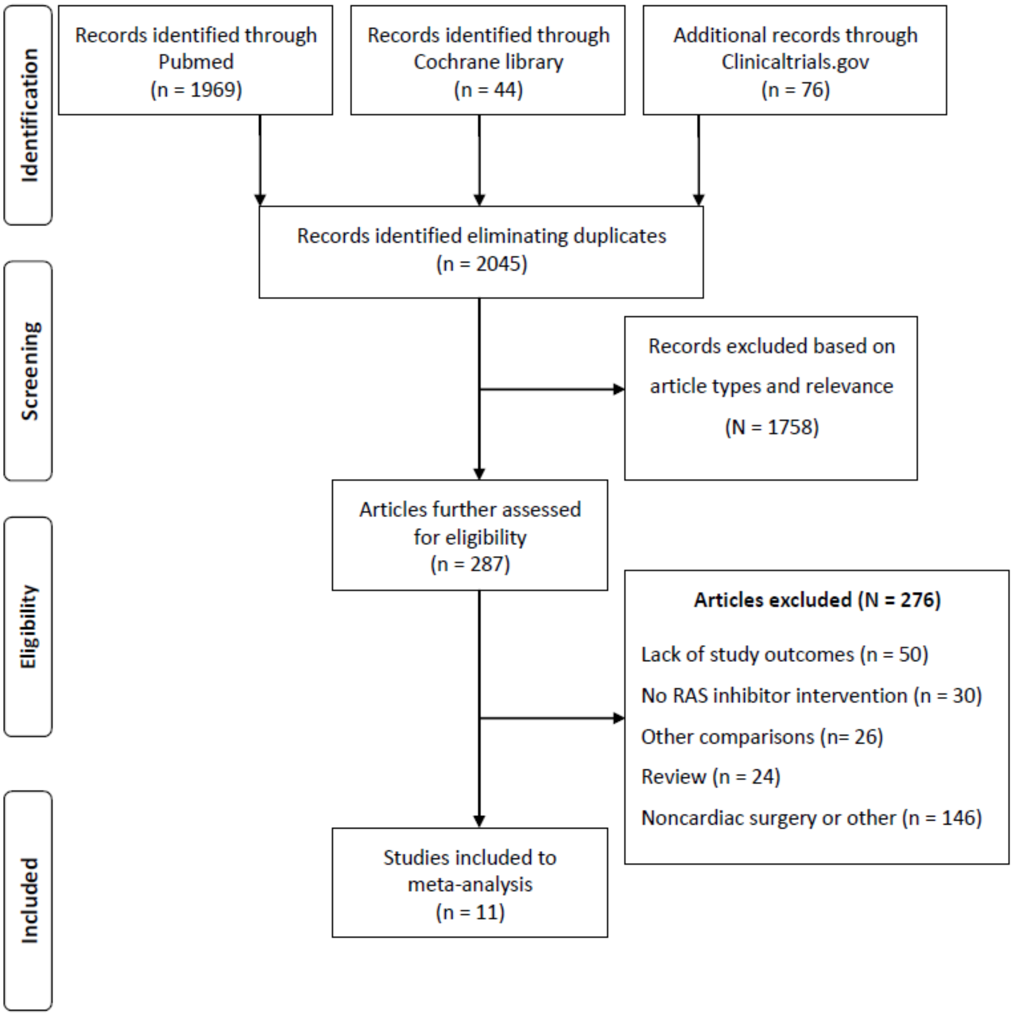

PRISMA: stands for Preferred Reporting Items for Systematic Reviews and Meta-Analyses, from The PRISMA statement for reporting systematic reviews and meta-analysis of studies that evaluate healthcare interventions: explanation and elaboration. BMJ 2009;339:b2700.

©2019 Chen S et al. *JAMA Network Open*

eTable 1. Quality Assessment

**Study reference**	**Prospective randomized trial?**	**Double blinded?**	**Clear definition of study population?**	**Clear definition of study comparison?**	**Clear definition of outcomes assessment?**	**Appropriate statistical method used?**	**No selective loss of data analysis?**	**Important confounders identified?**	**Quality Score**
White 2007[1]	No	No	Yes	Yes	Yes	Yes	Yes	Yes	6
Ozaydin2008[2]	Yes	No	Yes	Yes	Yes	Yes	Yes	Yes	7
Miceli 2009[3]	No	No	Yes	Yes	Yes	Yes	Yes	Yes	6
Rader 2010[4]	No	No	Yes	Yes	Yes	Yes	Yes	Yes	6
Yoo 2010[5]	No	No	Yes	Yes	Yes	Yes	Yes	Yes	6
El-Haddad2011[6]	Yes	No	Yes	Yes	Yes	Yes	Yes	Yes	7
Radaelli 2011[7]	No	No	Yes	Yes	Yes	Yes	Yes	Yes	6
Barodka 2011[8]	No	No	Yes	Yes	Yes	Yes	Yes	Yes	6
Bandeali 2012[9]	No	No	Yes	Yes	Yes	Yes	Yes	Yes	6
Chin 2012[10]	No	No	Yes	Yes	Yes	Yes	Yes	Yes	6
Pretorius 2012[11]	Yes	Yes	Yes	Yes	Yes	Yes	Yes	Yes	8

©2019 Chen S et al. *JAMA Network Open*

eTable 2 Summary of Outcome Data Reported in Included Studies

Study reference	Sample size (n)	AF events (n)	Stroke (n)	Death (n)	Composite outcome (n)	ICU or (hospital) stay (days)
A. White 2007[1]	175/163	51/59	NA	NA	51/59	NA
B. Ozaydin 2008[2]	98/30	11/10	NA	NA	11/10	NA
C. Miceli 2009[3]	3052/3052	763/610	21/31	40/20	824/661	NA
D. Rader 2010[4]	3437/3437	1023/992	46/61	47/51	1116/1104	NA
E. Yoo 2010[5]	296/176	61/37	NA	1/2	62/39	(12.6±7.9/11.4±4.2)
F. El-Haddad 2011[6]	50/50	3/11	NA	NA	3/11	2.9±1.3/3.5±1.7
G. Radaelli 2011[7]	1635/1504	342/335	NA	168/141	510/476	NA
H. Barodka 2011[8]	122/224	69/122	5/14	7/9	81/145	5.1±8.8/4.7±6.4
I. Bandeali 2012[9]	3983/4906	1062/1166	123/125	144/199	1329/1490	NA
J. Chin 2012[10]	407/643	77/94	NA	NA	77/94	NA
K. Pretorius 2012[11]	298/147	80/40	5/4	3/2	88/46	(5.8±3.3/6.8±8.2)

/: RASI / control group, ICU: intensive care unit, NA: not available.

©2019 Chen S et al. *JAMA Network Open*

eTable 3. Use of RASIs in Individual Studies

Study	RASI initiation	Agents	Dosage
A. White 2007[1]	RAS inhibitors preoperative	ACEI/ARB	Physician's discretion
B. Ozaydin 2008[2]	RAS inhibitors Preoperative at least 1 month	ACEI/ARB	Physician's discretion
C. Miceli 2009[3]	ACEI preoperative	ACEI	Physician's discretion
D. Rader 2010[4]	RAS inhibitors preoperative	ACEI/ARB	Physician's discretion
E. Yoo 2010[5]	RAS inhibitors Preoperative 2 weeks	ACEI/ARB	Physician's discretion
F. El-Haddad 2011[6]	ACEI/ARB preoperative at least 5 days	ARB: irbesartan	75 or 150 mg/day, without hemodynamic instability
G. Radaelli 2011[7]	RAS inhibitors Preoperative at least 2 weeks	ACEI/ARB	Physician's discretion
H. Barodka 2011[8]	RAS inhibitors preoperative	ACEI/ARB	Physician's discretion
I. Bandeali 2012[9]	ACEI preoperative	ACEI	Physician's discretion
J. Chin 2012[10]	RAS inhibitors preoperative	ACEI/ARB	Physician's discretion
K. Pretorius 2012[11]	RAS inhibitors Preoperative at least 4-7 days	RASI	Ramipril 2.5-5mg/day; Spironolactone 25mg/day

©2019 Chen S et al. *JAMA Network Open*

eTable 4. Concomitant AADs Use

Study	Sample size (n)	I/III AADs (%)
A. White 2007[1]	175/ 163	Preoperative amiodarone 38.3/ 37.4
B. Ozaydin 2008[2]	98/ 30	Discretion of the physician
C. Miceli 2009[3]	3052/ 3052	Not used
D. Rader 2010[4]	3437/ 3437	Not used
E. Yoo 2010[5]	296/ 176	Not used
F. El-Haddad 2011[6]	50/ 50	Not used
G. Radaelli 2011[7]	1635/ 1504	Not used
H. Barodka 2011[8]	122/ 224	Not used
I. Bandeali 2012[9]	3983/ 4906	Not used
J. Chin 2012[10]	407/ 643	Not used
K. Pretorius 2012[11]	298/ 147	Not used

AADs: antiarrhythmic drugs

eFigure 2. Sensitivity Analysis

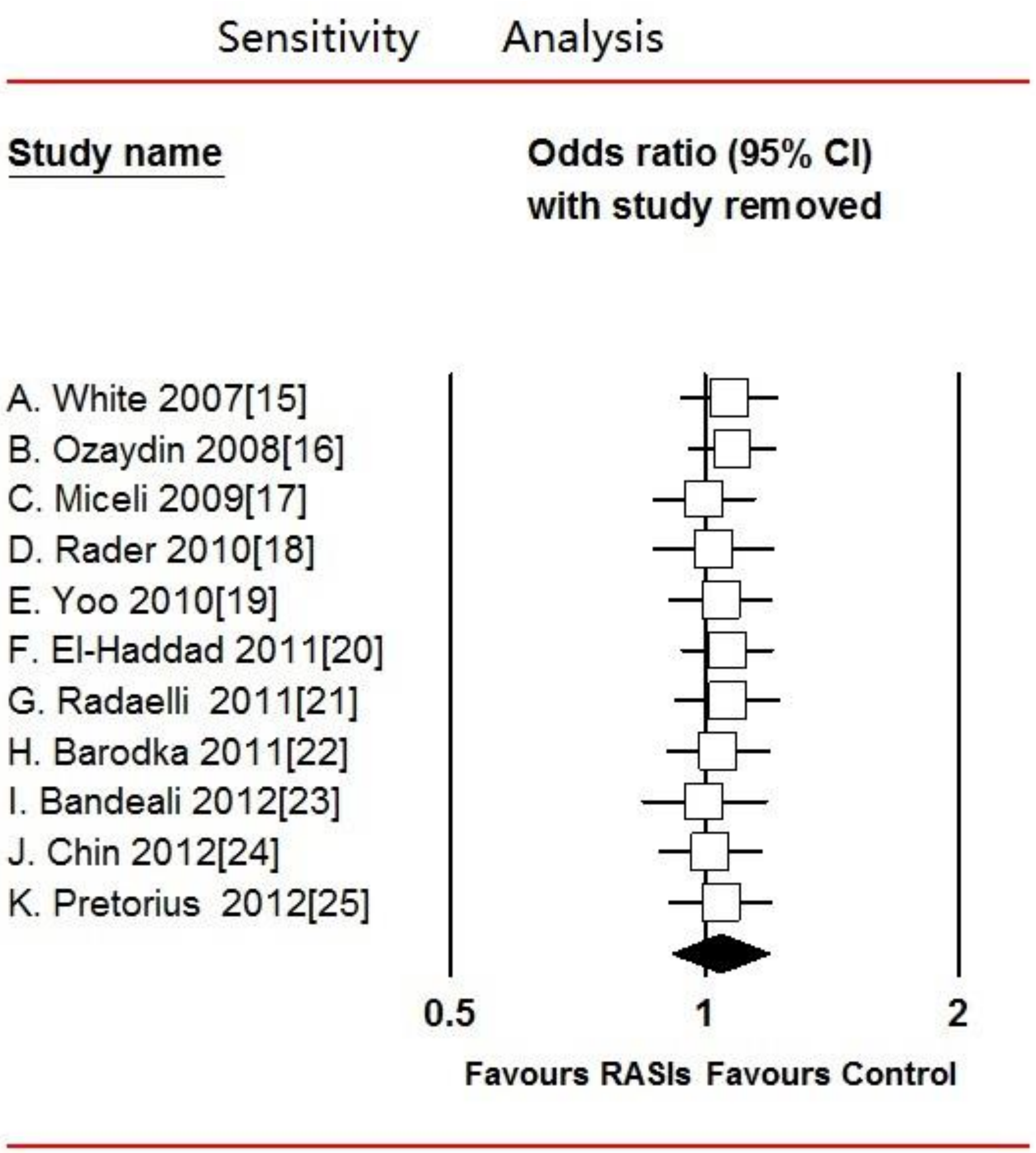

©2019 Chen S et al. *JAMA Network Open*

eFigure 3. Comparison for Risk of Stroke, Mortality, and Composite Events

Pooled -analysis for the comparison of risk of postoperative stroke, death, and composite adverse cardiac events

A: Comparison for risk of stroke

	RAS inhibitors		Control			Odds Ratio
Study or Subgroup	Events	Total	Events	Total	Weight	M-H, Random, 95% CI
C. Miceli. 2009	21	3052	31	3052	20.1%	0.68 [0.39, 1.18]
D. Rader. 2010	46	3437	61	3437	28.9%	0.75 [0.51, 1.10]
H. Barodka. 2011	5	122	14	224	8.1%	0.64 [0.23, 1.82]
I. Bandeali. 2012	123	3983	125	4906	37.5%	1.22 [0.95, 1.57]
K. Pretorius. 2012	5	298	4	147	5.4%	0.61 [0.16, 2.31]
**Total (95% CI)**		**10892**		**11766**	**100.0%**	**0.86 [0.62, 1.19]**
Total events	200		235			

Heterogeneity: Tau² = 0.06; Chi² = 7.54, df = 4 (P = 0.11); I² = 47%
Test for overall effect: Z = 0.90 (P = 0.37)

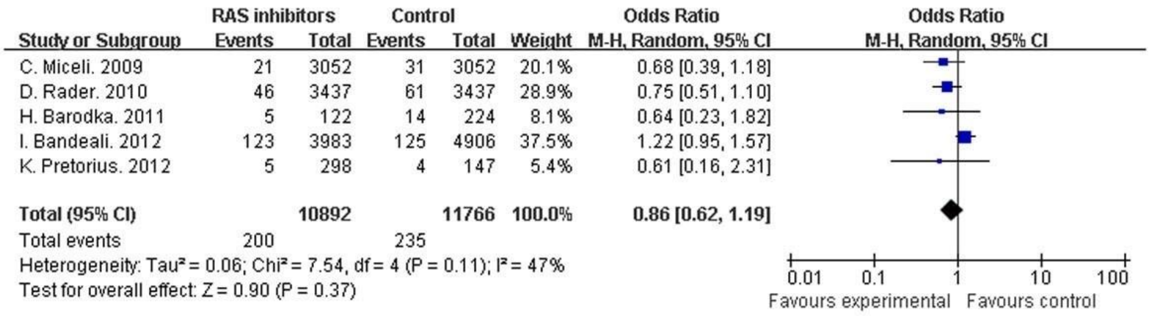

B: Comparison for risk of death

	RAS inhibitors		Control			Odds Ratio
Study or Subgroup	Events	Total	Events	Total	Weight	M-H, Random, 95% CI
C. Miceli. 2009	40	3052	20	3052	12.8%	2.01 [1.17, 3.45]
D. Rader. 2010	47	3437	51	3437	18.8%	0.92 [0.62, 1.37]
E. Yoo. 2010	1	296	2	176	0.9%	0.29 [0.03, 3.28]
G. Radaelli. 2011	168	1635	141	1504	30.0%	1.11 [0.87, 1.40]
H. Barodka. 2011	7	122	9	224	4.6%	1.45 [0.53, 4.01]
I. Bandeali. 2012	144	3983	199	4906	31.4%	0.89 [0.71, 1.10]
K. Pretorius. 2012	3	298	2	147	1.6%	0.74 [0.12, 4.46]
**Total (95% CI)**		**12823**		**13446**	**100.0%**	**1.07 [0.85, 1.35]**
Total events	410		424			

Heterogeneity: Tau² = 0.03; Chi² = 10.00, df = 6 (P = 0.12); I² = 40%
Test for overall effect: Z = 0.59 (P = 0.56)

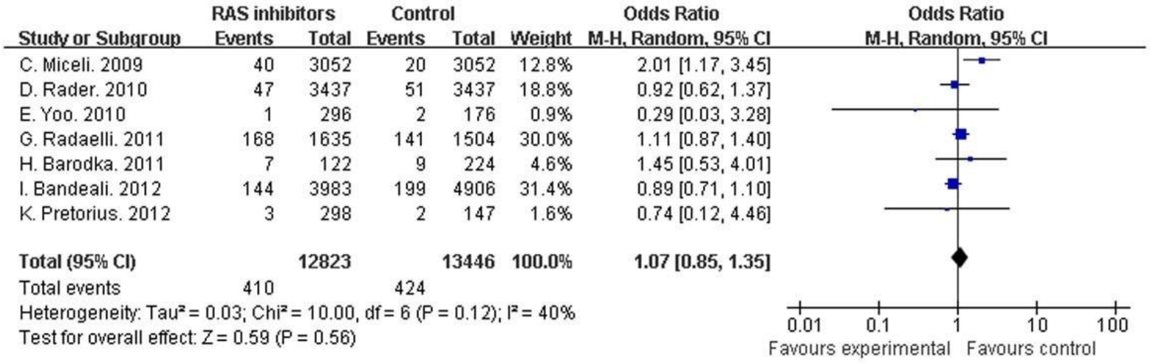

C: Comparison for risk of composite adverse cardiac events

	RAS inhibitors		Control			Odds Ratio
Study or Subgroup	Events	Total	Events	Total	Weight	M-H, Random, 95% CI
A. White. 2007	51	175	59	163	5.8%	0.72 [0.46, 1.14]
B. Ozaydın. 2008	11	98	10	30	1.6%	0.25 [0.09, 0.68]
C. Miceli. 2009	824	3052	661	3052	16.3%	1.34 [1.19, 1.50]
D. Rader. 2010	1116	3437	1104	3437	16.9%	1.02 [0.92, 1.12]
E. Yoo. 2010	62	296	39	176	5.8%	0.93 [0.59, 1.46]
F. El-Haddad. 2011	3	50	11	50	0.9%	0.23 [0.06, 0.87]
G. Radaelli. 2011	510	1635	476	1504	15.0%	0.98 [0.84, 1.14]
H. Barodka. 2011	81	122	145	224	5.6%	1.08 [0.68, 1.71]
I. Bandeali. 2012	1329	3983	1490	4906	17.2%	1.15 [1.05, 1.26]
J. Chin. 2012	77	407	94	643	8.6%	1.36 [0.98, 1.90]
K. Pretorius. 2012	88	298	46	147	6.3%	0.92 [0.60, 1.41]
**Total (95% CI)**		**13553**		**14332**	**100.0%**	**1.04 [0.91, 1.18]**
Total events	4152		4135			

Heterogeneity: Tau² = 0.02; Chi² = 36.02, df = 10 (P < 0.0001); I² = 72%
Test for overall effect: Z = 0.56 (P = 0.58)

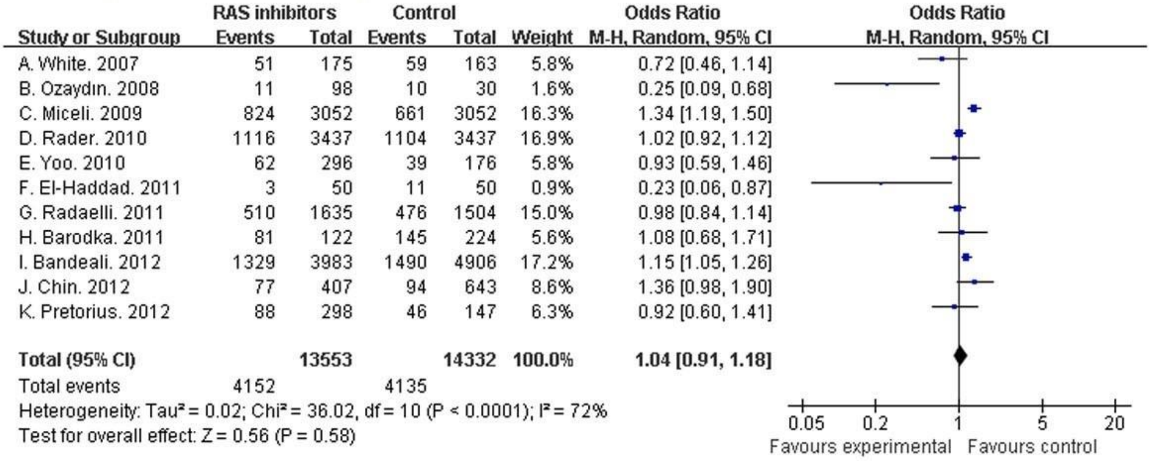

©2019 Chen S et al. *JAMA Network Open*

eFigure 4. Comparison for Hospitalization

	RAS inhibitors			Control				Mean Difference
**Study or Subgroup**	**Mean**	**SD**	**Total**	**Mean**	**SD**	**Total**	**Weight**	**IV, Random, 95% CI**
E. Yoo. 2010	12.6	7.9	296	11.4	4.2	176	26.4%	1.20 [0.11, 2.29]
F. El-Haddad. 2011	2.9	1.3	50	3.5	1.7	50	33.7%	-0.60 [-1.19, -0.01]
H. Barodka. 2011	5.1	8.8	122	4.7	6.4	224	17.6%	0.40 [-1.37, 2.17]
K. Pretorius. 2012	5.8	3.3	298	6.8	8.2	147	22.4%	-1.00 [-2.38, 0.38]
**Total (95% CI)**			**766**			**597**	**100.0%**	**-0.04 [-1.05, 0.98]**

Heterogeneity: Tau² = 0.70; Chi² = 9.75, df = 3 (P = 0.02); I² = 69%
Test for overall effect: Z = 0.07 (P = 0.94)

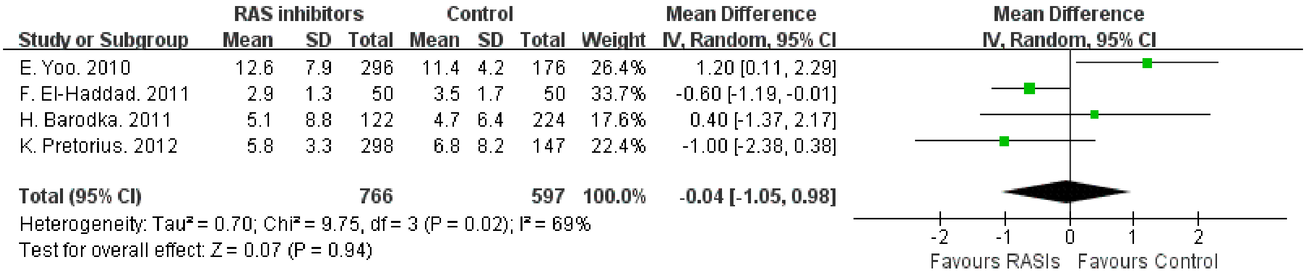

©2019 Chen S et al. *JAMA Network Open*

eReferences.

1. White CM, Kluger J, Lertsburapa K, Faheem O, Coleman CI. Effect of preoperative angiotensin converting enzyme inhibitor or angiotensin receptor blocker use on the frequency of atrial fibrillation after cardiac surgery: a cohort study from the atrial fibrillation suppression trials II and III. Eur J Cardiothorac Surg. 2007;31:817-20.
2. Ozaydin M, Dede O, Varol E, Kapan S, Turker Y, Peker O, et al. Effect of renin-angiotensin aldosteron system blockers on postoperative atrial fibrillation. Int J Cardiol. 2008;127:362-7.
3. Miceli A, Capoun R, Fino C, Narayan P, Bryan AJ, Angelini GD, et al. Effects of angiotensin-converting enzyme inhibitor therapy on clinical outcome in patients undergoing coronary artery bypass grafting. J Am Coll Cardiol. 2009;54:1778-84.
4. Rader F, Van Wagoner DR, Gillinov AM, Blackstone EH. Preoperative angiotensin-blocking drug therapy is not associated with atrial fibrillation after cardiac surgery. Am Heart J. 2010 ;160:329-336.
5. Yoo YC, Youn YN, Shim JK, Kim JC, Kim NY, Kwak YL. Effects of renin-angiotensin system inhibitors on the occurrence of acute kidney injury following off-pump coronary artery bypass grafting. Circ J. 2010;74:1852-8.
6. El-Haddad MA, Zalawadiya SK, Awdallah H, Sabet S, El-Haddad HA, Mostafa A, et al. Role of irbesartan in prevention of post-coronary artery bypass graft atrial fibrillation. Am J Cardiovasc Drugs. 2011;11:277-84.
7. Radaelli G, Bodanese LC, Guaragna JC, Borges AP, Goldani MA, Petracco JB, et al. The use of inhibitors of angiotensin-converting enzyme and its relation to events in the postoperative period of CABG. Rev Bras Cir Cardiovasc. 2011;26:373-9.
8. Barodka V, Silvestry S, Zhao N, Jiao X, Whellan DJ, Diehl J, et al. Preoperative renin-angiotensin system inhibitors protect renal function in aging patients undergoing cardiac surgery. J Surg Res. 2011;167:e63-9.
9. Bandeali SJ, Kayani WT, Lee VV, Pan W, Elayda MA, Nambi V, et al. Outcomes of preoperative angiotensin-converting enzyme inhibitor therapy in patients undergoing isolated coronary artery bypass grafting. Am J Cardiol. 2012;110:919-23.
10. Chin JH, Lee EH, Son HJ, Kim WJ, Choi DK, Park SK, et al. Preoperative treatment with an angiotensin-converting enzyme inhibitor or an angiotensin receptor blocker has no beneficial effect on the development of new-onset atrial fibrillation after off-pump coronary artery bypass graft surgery. Clin Cardiol. 2012;35:37-42.
11. Pretorius M, Murray KT, Yu C, Byrne JG, Billings FT 4th, Petracek MR, et al. Angiotensin-converting enzyme inhibition or mineralocorticoid receptor blockade do not affect prevalence of atrial fibrillation in patients undergoing cardiac surgery. Crit Care Med. 2012;40:2805-12.

©2019 Chen S et al. *JAMA Network Open*
